# PhenoFECT: A Python package for forecasting and exploring plant phenology under climate change

**DOI:** 10.1002/aps3.70088

**Published:** 2026-10-30

**Authors:** Songwon Kim, In‐Geol Choi

**Affiliations:** ^1^ Division of Life Science, College of Life Sciences and Biotechnology Korea University Seoul 02481 Republic of Korea; ^2^ FablessBio Inc. and Department of Biotechnology, College of Life Sciences and Biotechnology Korea University Seoul 02481 Republic of Korea

**Keywords:** chill‐day model, climate change, climate change indicators, daylength, flowering date prediction, phenology shifts, plant phenology

## Abstract

**Premise:**

Climate change and global warming significantly affect the timing of plant flowering in spring. Accurately predicting these phenological events is crucial for understanding plant biology and agriculture. The chill‐day model (CDM) based on seasonal temperature changes has been a reliable model for predicting phenological activities; however, recent global warming has challenged its accuracy due to extreme weather variability, necessitating the adjustment of the CDM parameters to reflect recent climate change. We developed an open‐source Python package, PhenoFECT (Phenology Forecasting Exploring ClimaTe), that refines temperature‐based phenology models. The package enables efficient phenological analysis for open and user‐specified data.

**Methods and Results:**

The Python package embeds open meteorological data collected over 53 years (1973–2025) in Korea for cherry (*Prunus yedoensis*), golden‐bell (*Forsythia koreana*), and azalea (*Rhododendron mucronulatum*). PhenoFECT was utilized to adjust the parameters of the CDM to reflect recent climate changes. The refined CDM improved the prediction accuracy of flowering at the average root mean square error (RMSE) of 5.74 days for three angiosperms.

**Conclusions:**

PhenoFECT is a versatile tool for accurate forecasting, exploration, and visualization across diverse open meteorological data. Exploring data using PhenoFECT revealed that the CDM can be influenced by geographic features and global climate phenomena.

Since pre‐industrial times, the increasing atmospheric concentration of greenhouse gases such as CO_2_, CH_4_, and N_2_O has significantly altered the climate system, leading to global warming. With industrialization, human‐induced global warming is estimated to be approximately 1.0°C ± 0.2°C (Masson‐Delmotte et al., 2018). Plant phenology is a crucial indicator of global warming because shifts in flowering and leafing dates directly reflect changes in temperature (Parolini, 2022). In temperate regions of the Northern Hemisphere, such as Korea and Japan, earlier spring phenological events have been widely observed due to increasing temperatures (Lee and Lee, 2003; Doi and Katano, 2008). These phenological shifts can affect ecosystem functions, agricultural practices, and biological interactions, making plant phenology monitoring essential for tracking climate change impacts. For example, a steeper increasing trend (approximately 2.1°C ± 0.2°C between the periods 1922–1940 and 1981–2000) in the monthly mean temperature in the early months of the year (February, March) in a metropolitan area (Seoul, Korea) led to earlier blooming of spring flowers (Ho et al., 2006).

The accurate prediction of phenological events is essential not only for research in plant biology and agriculture, but also for influencing local economies, as it can determine the success of seasonal festivals. In 2014, an especially early blooming season caused confusion and disruption across South Korea, as the dates on which cherry trees (*Prunus yedoensis* Matsum.) bloomed differed by a week compared to the dates predicted by the phenology observation data from the Korea Meteorological Administration (KMA) (Lee et al., 2014). Beyond economic and societal concerns, the shifting phenological timing of spring‐blooming species serves as a good indicator that can reflect the unpredictable patterns of climate change. For these reasons, the continued monitoring and analysis of plant phenology is essential (Korea Meteorological Administration, 2016).

Temperate spring‐flowering woody plants, such as cherry (*Prunus yedoensis*), golden‐bell (*Forsythia koreana* Nakai), and azalea (*Rhododendron mucronulatum* Turcz.), are suitable model species for capturing shifts in the climate patterns of the Northern Hemisphere (Ho et al., 2006). These model plants undergo a series of physiological changes such as floral differentiation, dormancy, budburst, and blooming as the season progresses. Temperature change is known to be the most critical factor in the various meteorological variables that regulate phenological events (Oh et al., 2004). The chill‐day model (CDM) is the most popular and widely used plant phenology model. It uses the “thermal time” unit, which reflects both temperature and time to predict the blooming process of spring flowers (Cesaraccio et al., 2004). Temperature‐based models such as the Utah model (Richardson et al., 1974) are classical and widely used, but the Utah model has proven inaccurate when evaluated under climate conditions different from those of the original experiments (Gilreath and Buchanan, 1981). The CDM is a general methodology that allows site‐ and species‐specific phenological modeling for diverse and changing climatic conditions. It is based on long‐term phenological observations of plant species and estimates three key parameters: temperature threshold (*T*_*c*_), chilling requirement (*C*_*r*_), and heat requirement (*H*_*r*_). These parameters enable the prediction of blooming dates solely from reliable temperature data.

Korea is in the temperate region of the Northern Hemisphere (33–43°N, 123–132°E) on a peninsula surrounded by the sea on three sides. Springtime flowering events progress from south to north, throughout regions with diverse geographic features, including urban, mountainous, and coastal regions (Lee and Lee, 2003). We further distinguished inland regions from island regions (e.g., observatory stations at Ulleungdo, Jeju, Seogwipo, and Seongsan) to account for their distinct climatic conditions. In Korea, the prediction of phenological events (e.g., flowering date) using the CDM was relatively more accurate than using regression analysis based on the average February and March temperatures from the KMA (Jung et al., 2005). The typical prediction error (root mean square error [RMSE]) of the CDM is around two days in Korea (Jung et al., 2005), but the error is likely to be increasing every year due to climate change. The flowering dates of areas without previous phenological observation data can be predicted using flowering distribution data collected in neighboring regions (Kim et al., 2013). The inverse distance weighting (IDW) method was applied to regions categorized by topographical characteristics, resulting in a best‐case average prediction error of less than three days, but the prediction error increases with increasing proximity to coastal and southern regions (Kim et al., 2013). Because the CDM predicts plant phenology based on temperature data, we hypothesized that regions with similar climatic conditions may exhibit similar modeled phenological patterns. Based on this hypothesis, we conducted additional analyses such as cluster analysis to classify distinct climatic patterns associated with modeling difficulties.

To survey and handle how the predictive power of phenological models responds to recent climate change, including rapidly rising temperatures, we developed an open‐source Python package called Phenology Forecasting and Exploring under ClimaTe change (PhenoFECT). This package is a multifunctional tool that enables researchers to predict the responses of phenological events under climate change by refining plant phenology models. PhenoFECT embeds Korean (1904–2025) and Japanese (1976–2025) open meteorological data for analyzing climate patterns at regional or national scales. The package provides a visualization of analytical results for predicted flowering dates of model plants. To increase its prediction power of flowering dates, PhenoFECT was utilized to adjust the CDM parameters reflecting recent climatic changes. Using the refined CDM parameters, PhenoFECT achieved the highest prediction accuracy for the flowering phenological events of three temperate spring‐flowering woody plants than previous reports using the RMSE. PhenoFECT suggests new potential parameters that could improve the accuracy of models under specific weather conditions, such as mild winters.

## METHODS AND RESULTS

PhenoFECT is an open‐source Python package written in Python 3.10. The source code, detailed user manual, and embedded dataset are available at https://github.com/SongWon03/PhenoFECT (see Data Availability Statement). The released package on Python Package Index (PyPI) can be easily installed via the pip package manager. When installing the package, the embedded dataset containing meteorological and phenological observations from Korea and Japan in comma‐separated values (CSV) format is also downloaded and can be readily used within the package.

### Input preparation

The required input for PhenoFECT includes meteorological data with daily temperature observations and phenological data for target species in CSV format (Figure 1). In the embedded dataset, we obtained phenological data for cherry (*Prunus yedoensis*), golden‐bell (*Forsythia koreana*), and azalea (*Rhododendron mucronulatum*) from public data sources provided by the KMA (Korea Meteorological Administration, 2025c). This phenological data includes budburst and flowering dates recorded at 95 meteorological stations across South Korea from 1973 to 2025. Budburst is defined as the stage at which approximately 20% of the total buds have burst, with visible leaves or petals. The flowering date is recorded when at least three blossoms have fully opened on a branch (Korea Meteorological Administration, 2016). The columns “bud_burst_date” and “flowering_date” represent the observation dates of the respective phenological events, while the columns “bud_burst_days” and “flowering_days” indicate the number of days elapsed since January 1 of the corresponding year (i.e., day of year (DOY) – 1), calculated for computational convenience. To predict phenological events using the CDM, we also collected daily meteorological data for the same 95 regions from 1904 to 2025 (Korea Meteorological Administration, 2025a). Temperature observations were categorized into daily average (“avgTa” column), minimum (“minTa”), and maximum (“maxTa”) values. The embedded dataset also contains the flowering phenological data of six angiosperm species recorded at 102 locations in Japan from 1953 to 2024 provided by the Japan Meteorological Agency (JMA); daily meteorological data from 1976 to 2025 for 101 regions in Japan was collected using the R package jmastats (Uryu, 2024).

**Figure 1:**
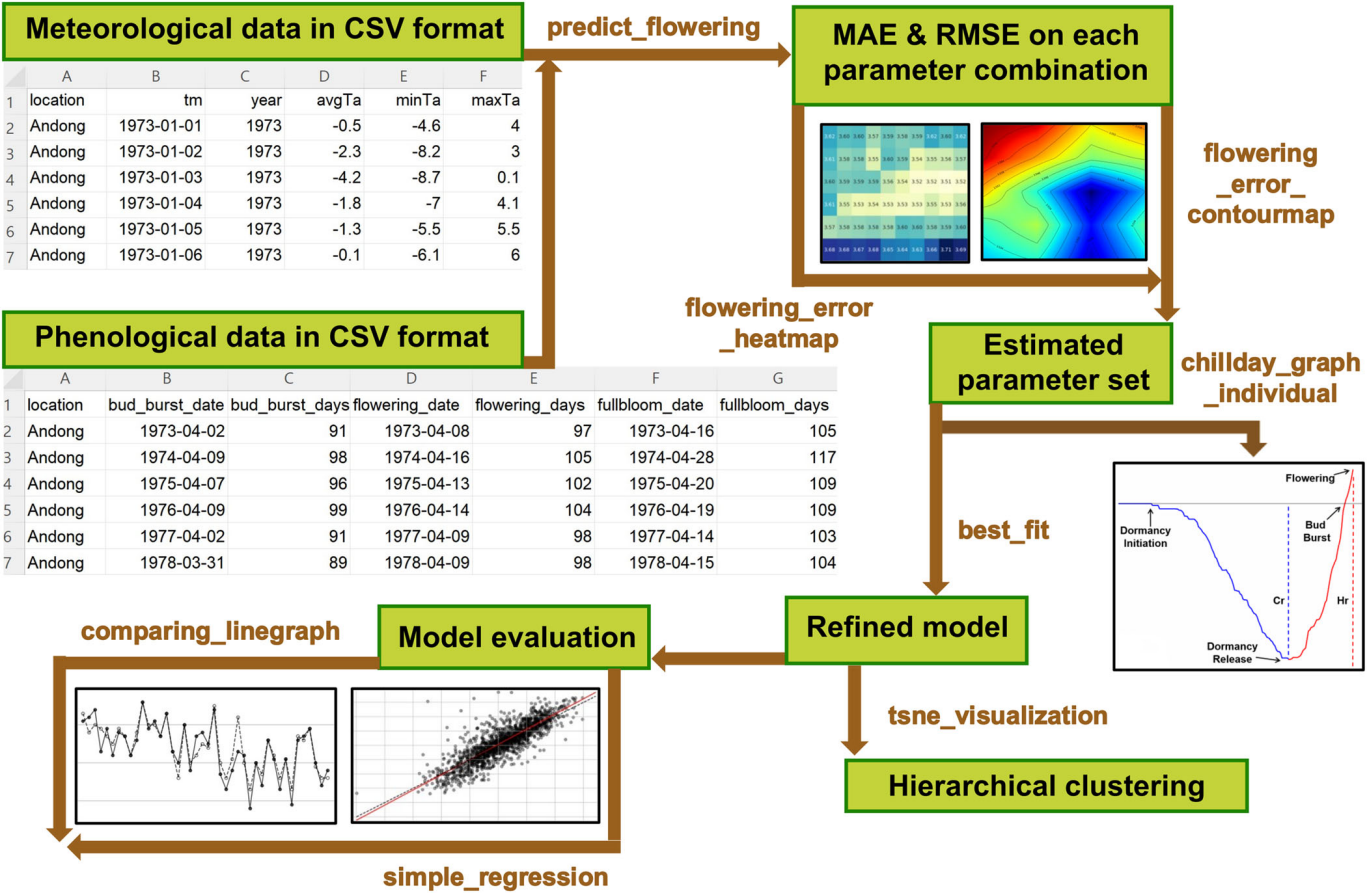
The PhenoFECT workflow. PhenoFECT processes the meteorological and phenological input data (in CSV format) through eight main functions: *predict_flowering*, *flowering_error_heatmap*, *flowering_error_contourmap*, *chillday_graph_individual*, *best_fit*, *comparing_linegraph*, *simple_regression*, and *tsne_visualization*. These functions enable prediction, model evaluation, and the generation of informative plots that are useful throughout the phenological analysis process.

To utilize PhenoFECT for various plant species, the user needs to prepare input data that matches the column names and data structures specified in the embedded dataset (Figure 1). Temperature observations should be from the same regional scale at which the phenological observations of the user‐specified plant species were recorded.

### Parameter estimation of the chill‐day model

The CDM is a sequential model that represents the physiological development of orchard crops and deciduous forest trees over thermal time (i.e., a combination of temperature and time). The underlying idea is that sufficient chilling accumulation during winter breaks the rest and heating accumulation in spring overcomes quiescence (Cesaraccio et al., 2004). The model was originally designed to predict budburst; it has also demonstrated reliable accuracy in forecasting blooming dates (Kim et al., 2013).

Although phenological events follow a regular seasonal cycle, their timing according to the calendar date changes each year, as illustrated in the CDM (Figure 2). Dormancy initiation of temperate zone deciduous trees starts from the day when the minimum temperature falls below the *T*_*c*_. Thereafter, the amount of chilling and heating is calculated daily using CDM formulas (Cesaraccio et al., 2004), which are divided into six cases based on how the daily maximum (*T*_*x*_), minimum (*T*_*n*_), and mean (*T*_*m*_) temperatures relate to the *T*_*c*_ value (Table 1). Chill days (*C*_*d*_) and anti‐chill days (*C*_*a*_) represent thermal time through the daily accumulation of chilling and heating, respectively, used by temperate deciduous tree species and orchard crops to regulate seasonal development. They undergo endodormancy until accumulated *C*_*d*_ fall below the sufficient amount of cold (i.e., *C*_*r*_). This mechanism prevents flowering in winter due to temporary warmth. After release from endodormancy, these plants enter environmental dormancy. Phenological events such as budburst and blooming occur when the accumulated *C*_*a*_ reach *H*_*r*_. Because budburst and flowering require different amounts of heat, *H*_*r*_ must be estimated separately for each event. For budburst prediction, *H*_*r*_ is defined as the absolute value of *C*_*r*_.

**Figure 2:**
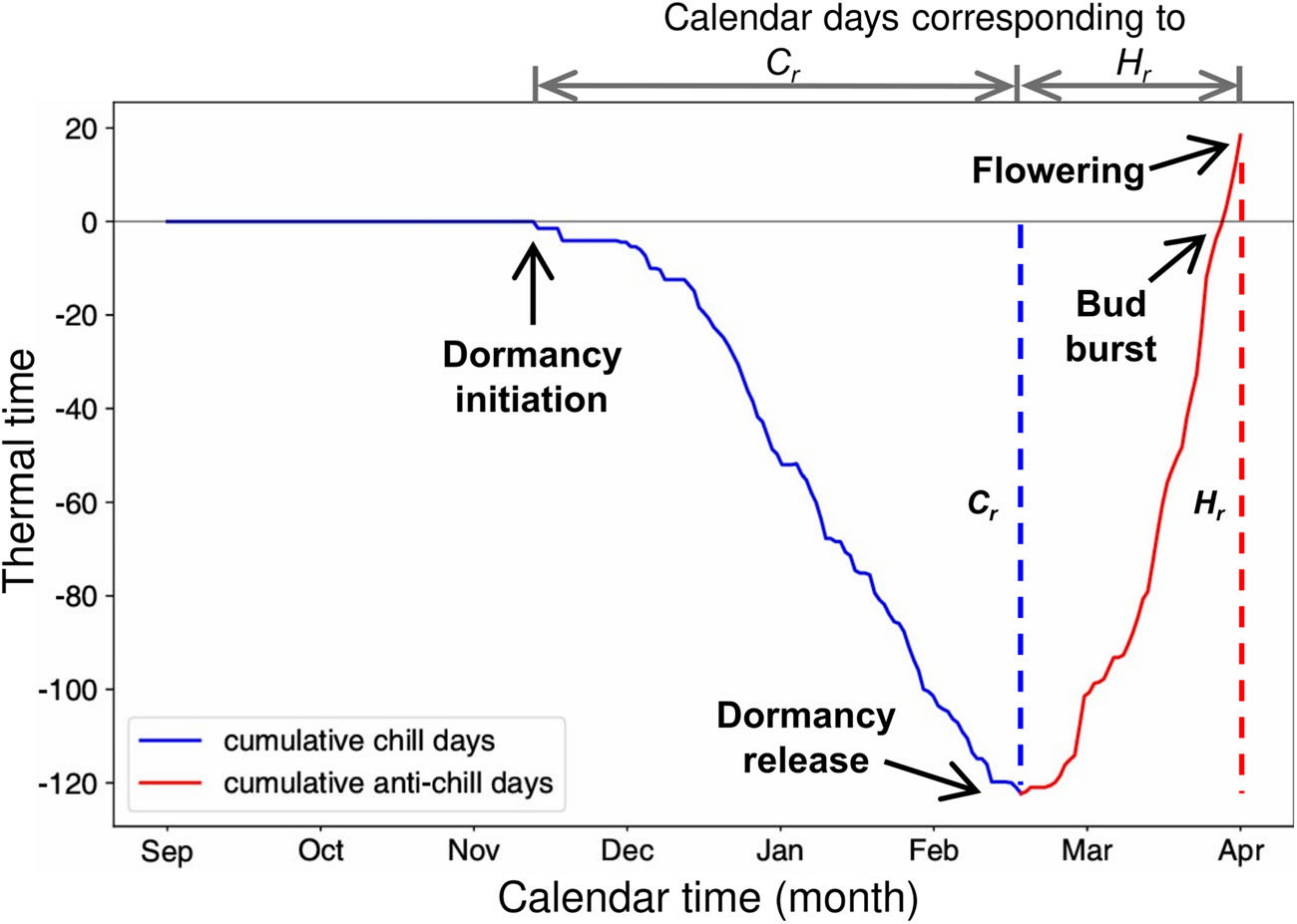
Accumulation of chill days (*C*_*d*_) and anti‐chill days (*C*_*a*_) represented as thermal time. Temperate zone deciduous trees undergo seasonal changes in response to sequential exposure to long periods of chilling and warming. Dormancy is released when the cumulative chill days reach the chilling requirement (*C*_*r*_) and the sufficient accumulation of anti‐chill days beyond the heat requirement (*H*_*r*_) induces budburst and flowering events.

**Table 1:** Chill days (*C*_*d*_) and anti‐chill days (*C*_*a*_) equations for the six temperature scenarios.

Case	Temperature cases	Chill days (*C*_*d*_)	Anti‐chill days (*C*_*a*_)
1	0 ≤ *T*_*c*_ ≤ *T*_*n*_ ≤ *T*_*x*_	*C*_*d*_ = 0	*C*_*a*_ = *T*_*m*_ – *T*_*c*_
2	0 ≤ *T*_*n*_ ≤ *T*_*c*_ < *T*_*x*_	*C*_*d*_ = – [(*T*_*m*_ – *T*_*n*_) – (*T*_*x*_ – *T*_*c*_)^2^/2(*T*_*x*_ – *T*_*n*_)]	*C*_*a*_ = (*T*_*x*_ – *T*_*c*_)^2^/2(*T*_*x*_ – *T*_*n*_)
3	0 ≤ *T*_*n*_ ≤ *T*_*x*_ ≤ *T*_*c*_	*C*_*d*_ = – (*T*_*m*_ – *T*_*n*_)	*C*_*a*_ = 0
4	*T*_*n*_ < 0 ≤ *T*_*x*_ ≤ *T*_*c*_	*C*_*d*_ = – [*T*_*x*_^2^/2(*T*_*x*_ – *T*_*n*_)]	*C*_*a*_ = 0
5	*T*_*n*_ < 0 < *T*_*c*_ < *T*_*x*_	*C*_*d*_ = – *T*_*x*_^2^/2(*T*_*x*_ – *T*_*n*_) – (*T*_*x*_ – *T*_*c*_)^2^/2(*T*_*x*_ – *T*_*n*_)	*C*_*a*_ = (*T*_*x*_ – *T*_*c*_)^2^/2(*T*_*x*_ – *T*_*n*_)
6	*T*_*x*_ < 0	*C*_*d*_ = 0	*C*_*a*_ = 0

*Note*: *T*_*c*_, temperature threshold; *T*_*m*_, daily mean temperature; *T*_*n*_, daily minimum temperature; *T*_*x*_, daily maximum temperature.

The *predict_flowering* function of PhenoFECT estimates three key parameters (*T*_*c*_, *C*_*r*_, and *H*_*r*_) required to predict flowering dates of a target plant species. These parameters are species‐specific and must be estimated separately for each species. To identify a combination of parameters that best explain phenological events, the *predict_flowering* function computes the mean absolute error (MAE) or RMSE between observed and predicted flowering dates for all candidate parameter sets. The equations for MAE and RMSE are represented as:

$$\mathrm{MAE}=\frac{1}{n}\;\sum_{i=1}^{n}\left|y_{i}-{\hat{y}}_{i}\right|,\;\mathrm{RMSE}=\sqrt{\frac{1}{n}\;\sum_{i=1}^{n}{\left(y_{i}-{\hat{y}}_{i}\right)}^{2}}$$

where *n* is the size of the data, $y_{i}$ is the observed date of the phenological event, and ${\hat{y}}_{i}$ is the predicted date of the phenological event. Users provide two input datasets in CSV format, and the *predict_flowering* function calculates the MAE or RMSE for each candidate parameter combination. The most accurate parameter set can be readily selected through visualization using the *flowering_error_heatmap* and *flowering_error_contourmap* functions, in a process analogous to a grid search (Figure 1).

### Hierarchical clustering

We assume that the effects of climate change on a regional scale can be identified by classifying the CDM curves derived from meteorological data; this assumption is based on the rationale that regions with similar climate patterns exhibit similar curve shapes in the CDM. The phenological events of angiosperm species can be predicted using the CDM with the optimal parameter set, provided that daily temperature data are available. With the input meteorological data, the *tsne_visualization* function first calculates a vector representing the daily increment of thermal time until flowering for each year. These vectors are classified using agglomerative clustering.

Agglomerative hierarchical clustering is a technique that produces partitions by a series of successive fusions of *n* observations into groups (Everitt and Dunn, 2001), with the distance between two groups defined using single linkage, complete linkage, or Ward's method (Ward, 1963). Users can select the clustering method with the function argument *method* as ‘single’, ‘complete’, or ‘ward’ to deal with cases such as a chaining effect. After the vectors are classified based on distances in the dendrogram, the *tsne_visualization* function projects these vectors into two‐ or three‐dimensional space and represents the relationship between the CDM curves (Figure 1). The *tsne_visualization* function is based on the t‐distributed stochastic neighbor embedding (t‐SNE) method, and users can visualize the reduced dimensional representation of vectors with function argument *n_components* as ‘2’ or ‘3’. In the *tsne_visualization* function, users can specify the ‘random_states’ and ‘perplexity’ parameters because t‐SNE is a stochastic dimensionality reduction method sensitive to these hyperparameters. Using a fixed ‘random_states’ parameter yields the same result for a given input dataset. Reproducibility can be evaluated by repeating the *tsne_visualization* function with multiple ‘random_states’ values. The ‘perplexity’ parameter controls the number of nearest neighbors considered during manifold learning. The recommended values of ‘perplexity’ range from 5 to 50 depending on the dataset size. In PhenoFECT, ‘random_states = 150’ and ‘perplexity = 50’ were used as default values to focus on the global pattern by considering a relatively large number of neighbors.

### Application of PhenoFECT to refine the chill‐day model for spring‐flowering plants in Korea

Utilizing the CDM to predict the flowering date of temperate spring‐blooming woody plants requires the proper estimation of three key parameters: *T*_*c*_, *C*_*r*_, and *H*_*r*_. We estimated separate parameters for each of three angiosperm species: cherry (*Prunus yedoensis*), golden‐bell (*Forsythia koreana*), and azalea (*Rhododendron mucronulatum*). Our goal was to evaluate how climate change affects the predictive power of the CDM and to refine it.

First, we selected 32, 31, and 26 locations from each phenological dataset where observations exist for more than 80% of the recorded years (1973–2025). The phenological dataset was then divided into two groups: training data for accurate parameter estimation and test data for analyzing prediction outcomes of models under changing climate conditions. Two years were randomly sampled from each decade starting from 1973 and used as test data. This random sampling procedure was performed three times to construct independent training–test splits for cross‐validation, enabling the estimation of generalizable CDM parameters to the overall time periods. Before predicting the flowering dates, we predicted budburst dates using the *predict_budburst* function to constrain the parameter space as a reference for parameter exploration aimed at flowering date prediction. With some outliers, the values of *T*_*c*_ vary from 7°C to 12°C and –100 to –200 chill days for *C*_*r*_ parameters (Cesaraccio et al., 2004). Taking into account the previously reported parameters for three angiosperm species (Kim et al., 2013), we tested 1200 combinations of *T*_*c*_ and *C*_*r*_ within the ranges of 2–8°C (in 1°C increments) and –50 to –200 (in 1‐unit increments), respectively. Based on the RMSE, the range of parameter combinations belonging to the first quartile (Q1) were selected for further examination using flowering date data from the training period. In the flowering date prediction stage, we started with a coarse resolution for testing CDM parameters of 1°C for the temperature threshold and three units for chill and heat requirements. Subsequently, a finer grid search was performed using resolutions of 0.1°C and one unit for the corresponding parameters for each of the three training datasets. The optimal parameter set for target angiosperm species was defined as the combination of CDM parameters with the minimum cross‐validation RMSE. Three test datasets were utilized to evaluate the average RMSE and select the optimal CDM parameters. The procedure for estimating the optimal parameter set using the functions *predict_flowering*, *flowering_error_heatmap*, and *flowering_error_contourmap* is presented in detail in Appendix S1 (see Supporting Information with this article), and sample codes are available on Github (https://github.com/SongWon03/PhenoFECT).

After the three key parameters of the CDM that yielded the minimum RMSE were selected separately for each model plant (Table 2), we evaluated whether the refined CDM parameters fit well on the overall time periods (1973–2025). The MAE and RMSE values show that the accuracy of the refined model improved substantially compared with the parameter set estimated from the 1951–1980 phenological observations in the previous study (Kim et al., 2013). In particular, the prediction error for cherry and golden‐bell trees was reduced by approximately one week, whereas the improvement for azalea was around one day (Table 2). This suggests that climate change such as rapid warming and temperature shifts significantly influences the predictive power of the CDM.

**Table 2:** Comparison of the accuracy of the refined chill‐day model and the estimated model from the previous study by Kim et al. (2013) for cherry, golden‐bell, and azalea.

Parameters and metrics	Cherry		Golden‐bell		Azalea	
	Refined	Previous	Refined	Previous	Refined	Previous
*T*_*c*_	3.9°C	7.0°C	3.2°C	6.0°C	2.8°C	6.0°C
*C*_*r*_	–108	–100	–81	–90	–68	–80
*H*_*r*_	164	158	132	128.5	158	94
MAE	3.74	11.91	4.96	11.25	5.66	6.59
RMSE	4.61	12.57	5.96	12.23	6.66	8.05

*Note*: *C*_*r*_, chilling requirement; *H*_*r*_, heat requirement; MAE, mean absolute error; RMSE, root mean square error; *T*_*c*_, temperature threshold.

Based on the refined model, users can evaluate the quality of prediction on a regional scale using the *comparing_linegraph* function (Appendix S2) or on a national scale using the *simple_regression* function (Figure 3). The overall performance of the temperature‐based phenology model is meaningful for real‐world applications such as agriculture. However, there are observatory stations where the RMSE value exceeds 10 days or the phenological events cannot be calculated due to specific climate conditions, for example, a mild winter.

**Figure 3:**
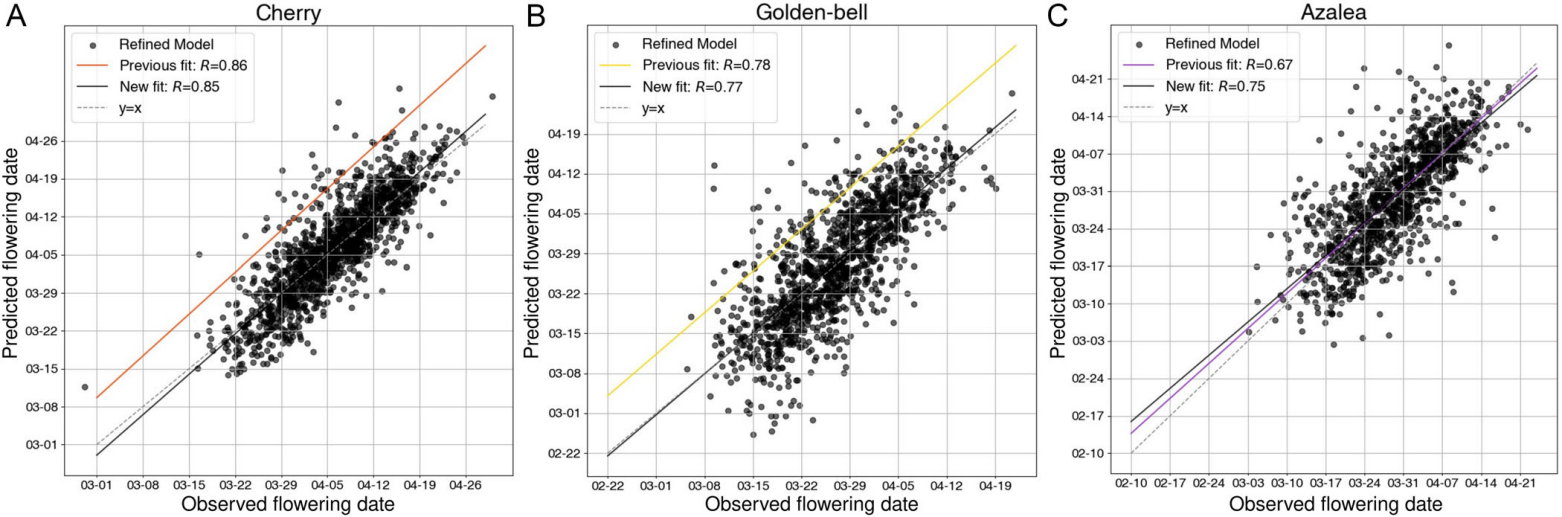
The relationship between the refined chill‐day model and the estimated model from the previous study by Kim et al. (2013) for predicted and observed flowering dates for cherry (A), golden‐bell (B), and azalea (C) at the 32, 31, and 26 phenology observatory stations of the Korea Meteorological Administration during 1973–2025. Colored solid lines (orange‐red, gold, dark orchid) indicate the regression lines based on the parameters estimated by Kim et al. (2013) using observations from 1951–1980, and black solid lines represent the refined regression lines. Dotted lines represent the 1:1 reference line (*y* = *x*), where predicted and observed flowering dates are equal.

### Enhancing performance with a new potential parameter

The observed phenological timing of the three plant species has advanced by approximately 10 days over the past 50 years (Figure 4). In particular, the distribution of flowering times has shifted drastically from 2014 in South Korea, and the difference between the earliest and latest flowering DOY has decreased to less than 20 days in the past eight years (Figure 4). Although the refined model reproduces the pattern of phenological events reasonably well with an RMSE of 5–6 days, there are difficulties in predicting the shifts relying solely on temperature data. Therefore, we examined ways to model the phenological events under climate change more precisely using meteorological variables with stable annual cycles.

**Figure 4:**
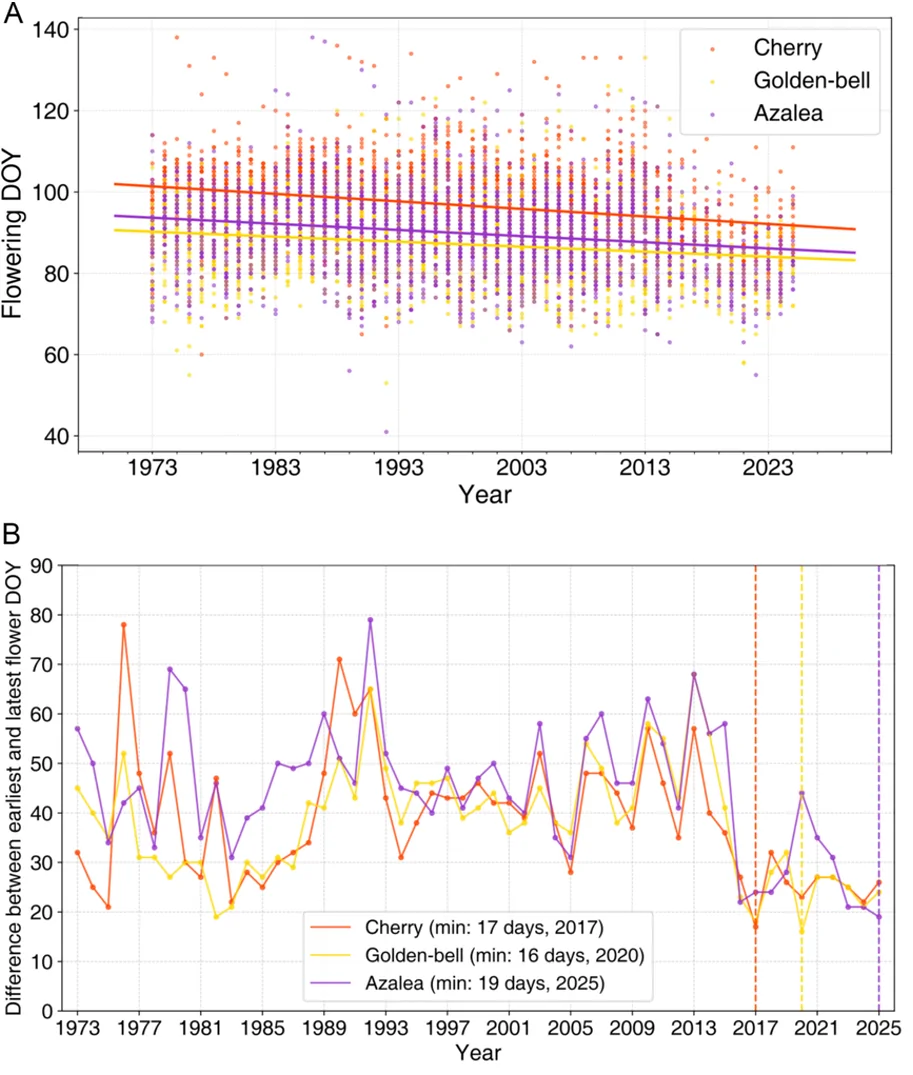
Shifts in the timing of flowering events of cherry, golden‐bell, and azalea (A), and the trend in the difference between the earliest and latest flowering day of year (DOY) among 95 observatory stations in South Korea (B) from 1973 to 2025. The best‐fit lines (solid lines; cherry, orange‐red; golden‐bell, gold; azalea, dark orchid) indicate that the phenological events of all three species have advanced in recent years (A). The difference between the earliest and latest flowering DOY has decreased markedly since 2014, with the minimum difference falling below 20 days during the past 10 years (B). The colored dotted lines show the year when the minimum difference was observed (cherry, 2017; golden‐bell, 2020; azalea, 2025).

Because long photoperiods may serve as an environmental cue to trigger physiological changes in plants under a mild winter (Körner and Basler, 2010), we introduced photoperiod as a new parameter to enhance the performance of the plant phenology model. Temperature‐only models are simple and rely on temperature, which is a relatively reliable variable. However, they are significantly affected by fluctuations and by a rapid increase in temperature. We expected that a meteorological variable such as photoperiod could serve as a complementary parameter due to the low variability of its cycle.

In South Korea, daylength decreases from approximately 12 to 9 h after dormancy initiation occurs, and then gradually increases back to around 12 h by the time spring‐flowering plants bloom. Accordingly, we modified the model to accumulate *C*_*a*_ if daylength exceeds the daylength threshold (*L*_*c*_). This allows the model to accumulate *C*_*a*_ even when *C*_*r*_ is not fulfilled under mild winter conditions. Although the proposed *L*_*c*_ represents a simplified implementation of photoperiod effects on dormancy release, we introduced it as a tunable, photoperiod‐related heuristic parameter inspired by experimental evidence.

While keeping the three key parameters, we examined the optimal value of *L*_*c*_ within the range of 10.1–13.5 h (in 0.1‐h increments) and then applied the extended parameter sets to the phenological data for the test period. In this case, phenological events of the three spring‐flowering plants were successfully predicted, except for the period when the temperature data were insufficient. Although the RMSE value at two coastal regions (Ulsan, Jinju) and three island observatory stations (Ulleungdo, Jeju, Seogwipo) exceeded 10 days, the overall performance of the refined model was enhanced (Table 3). Users can also estimate the *L*_*c*_ for user‐specified target plants using the *predict_flowering* function argument *day_length_list*.

**Table 3:** Enhanced performance of the refined chill‐day model considering photoperiod for cherry, golden‐bell, and azalea.

Parameters and metrics	Cherry		Golden‐bell		Azalea	
	Photoperiod	Refined	Photoperiod	Refined	Photoperiod	Refined
*L*_*c*_	11.3	None	11.1	None	11.2	None
MAE	3.63	3.74	4.8	4.96	5.29	5.66
RMSE	4.57	4.61	5.86	5.96	6.49	6.66

*Note*: *L*_*c*_ = daylength threshold; MAE = mean absolute error; RMSE = root mean square error.

### Exploring the prediction error trends using hierarchical clustering

Using the refined phenology model, we examined how seasonal conditions associated with climate change affect the predictive power of the model. This analysis was based on the idea that regions with similar climate patterns exhibit similar graph shapes in the CDM. We input the complete temperature data (1904–2025) into the *tsne_visualization* function, which calculated 2191 vectors representing phenological events for each observatory station and year, and performed agglomerative hierarchical clustering on these vectors. Users can readily prepare input data by specifying the range of years and clustering method in the *calculate_accumulation_application* function.

In particular, we analyzed cherry trees, which showed the highest sensitivity to rising temperatures. Because single linkage and complete linkage failed to separate clusters properly due to the chaining effect, Ward's method was applied. As a result, the hierarchical clustering classified the dataset into seven clusters excluding outliers (Figure 5). The *tsne_visualization* function automatically visualizes both individual clusters and the overall clustering results into two or three dimensions (Figure 5). Although dimensionality reduction with t‐SNE is sensitive to the ‘random_states’ and ‘perplexity’ parameters, the global pattern of visualization was almost reproduced with multiple hyperparameter combinations (Appendix S3). Clusters 4 and 9, in which the model predictions were much later or earlier than the actual phenological events, consisted of island observatory stations (Ulleungdo, Seongsan), northern coastal regions (Incheon, Sokcho), and southern coastal regions (Jinju, Mokpo, Ulsan). Overall, the prediction error of the refined model was higher in these regions than in urban and mountainous regions.

**Figure 5:**
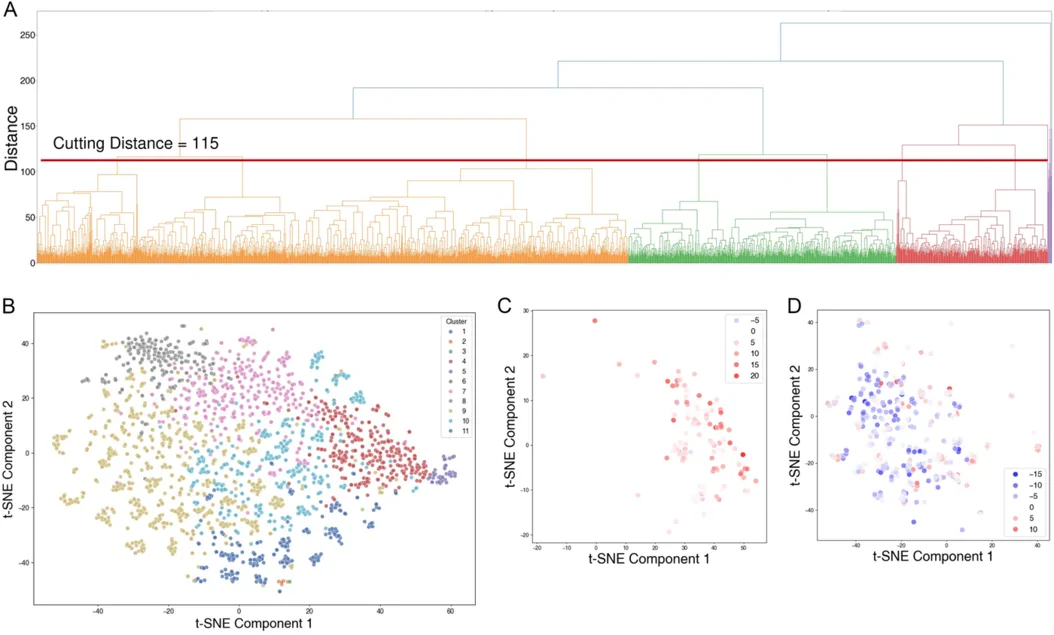
Output plots of the *tsne_visualization* function, including hierarchical clustering (A) and t‐distributed stochastic neighbor embedding (t‐SNE) visualizations (B–D). The *tsne_visualization* function uses ‘random_states = 150’ and ‘perplexity = 50’ as default values. The cutting distance for the dendrogram was selected manually by specifying the function argument ‘default_cutting = False’. With a cutting distance of 115, seven clusters were classified, excluding four clusters (clusters 1, 2, 3, and 6) that were considered outliers. Cluster 4 (red) and cluster 9 (gold) showed the trend of the most positive (C) and the most negative (D) prediction errors, respectively. The silhouette score was 0.014.

To examine the relationship between climate phenomena such as El Niño and La Niña and the predictive performance of the refined model, we obtained El Niño–Southern Oscillation (ENSO) occurrence year information from the KMA (Korea Meteorological Administration, 2025b). Based on the output CSV file generated by the *tsne_visualization* function, we tested whether ENSO events significantly affected the prediction accuracy of the temperature‐based model. The *prediction_error_shift* function plots the prediction errors of user‐assigned locations and performs a two‐sided permutation test (30,000 random reassignments) on yearly errors to compare El Niño or La Niña years against neutral years (Figure 6). Unlike the *predict_flowering* function, which calculates prediction errors using absolute values for MAE and RMSE estimation, the *tsne_visualization* function gives signed prediction errors in the output CSV file to enable ENSO‐related prediction error shift analysis.

**Figure 6:**
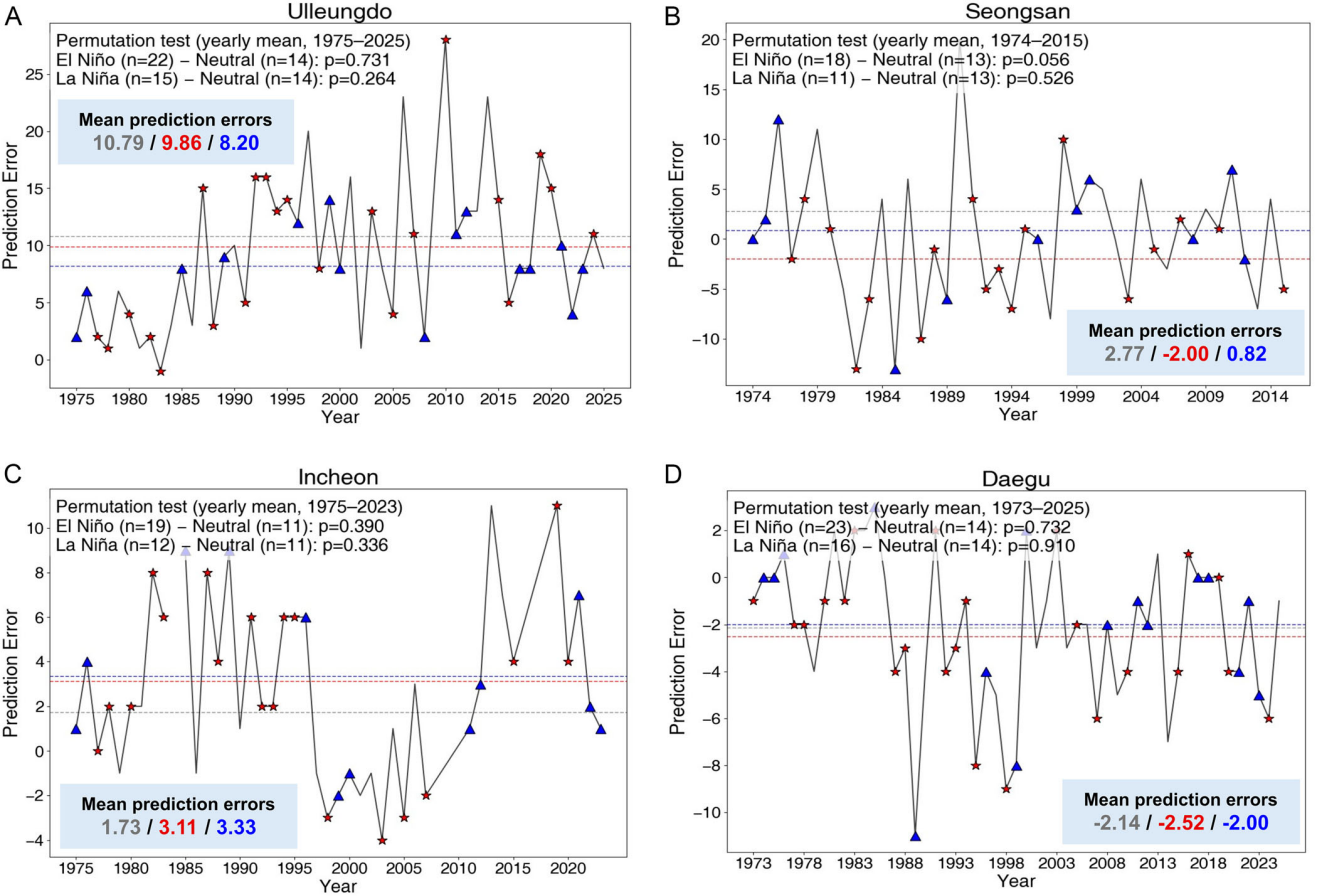
The influence of El Niño and La Niña events on the prediction errors of the temperature‐based model. The *prediction_error_shift* function was applied to four representative regions including island (A–B), coastal (C), and urban (D) areas. Yearly prediction errors were plotted, with El Niño (red stars) and La Niña (blue triangles) events highlighted. The dashed horizontal lines indicate the mean prediction error during non‐event (gray), El Niño (red), and La Niña (blue) years. A two‐sided permutation test (30,000 random reassignments) was conducted to evaluate whether the prediction errors during ENSO years significantly differed from those in neutral years. The mean difference in prediction error between ENSO years and neutral years was used as an effect size due to the limited number of samples. In the island region (Ulleungdo; A), a two‐sided permutation test revealed no significant differences when climate phenomena occurred (El Niño vs. neutral, *P* = 0.731; La Niña vs. neutral, *P* = 0.264), but the effect size for La Niña and neutral years was –2.59. No significant differences were detected on Seongsan, Jeju Island (B) and the coastal region (Incheon; C). However, the effect size for El Niño and neutral years in Seongsan was –4.77. The urban area (Daegu; D) showed no significant differences and effect size was more than –0.5.

Although the permutation test did not identify statistically significant prediction error shifts related to ENSO events in most regions, this may reflect the limited number of samples available for comparison. Therefore, we examined the effect size, defined as the mean difference in prediction error between ENSO and neutral years, to evaluate the practical magnitude of the shifts. Urban and mountainous regions generally showed small changes of less than one day for both ENSO years, whereas larger shifts exceeding 1–4 days were exhibited for the island and coastal regions. In most cases, the magnitude of the prediction error shift was greater during El Niño years than La Niña years (Figure 6). During climate phenomena phases, El Niño tends to change temperatures from winter to summer, which is critical for the physiology of flowering processes, whereas La Niña does not exhibit a consistent temperature trend but is associated with reduced precipitation (Cha et al., 1999). These results indicate that the accuracy of the CDM is sensitive to unexpected temperature variations. Additionally, Ulleungdo, the only region where the RMSE exceeds 10 days even with the refined model, was not significantly affected by ENSO events. This suggests that Ulleungdo has a unique geographic feature that is influenced by multiple meteorological factors beyond temperature, such as precipitation, humidity, and other local climate conditions.

## CONCLUSIONS

The timing of plant phenology such as flowering events is a crucial indicator of climate change, reflecting responses of life under global warming. To model phenological events and adjust CDM parameters under changing climatic conditions, we developed PhenoFECT, an open‐source and data‐embedded Python package for forecasting and exploring the phenology of diverse plants over recent climate change. Several tools have been available to facilitate the implementation of well‐established phenology models (Taylor, 2018; Luedeling et al., 2025) and to predict mean flowering dates of angiosperms even without observed climate scenarios (Park et al., 2019). However, PhenoFECT focuses on enabling CDM‐specific applications with extensive embedded East Asian data and an integrated exploration workflow (Table 4). PhenoFECT offers users a streamlined process that includes refining the parameters of the CDM for the target species, evaluating estimation quality, and examination of the predictive power of the model under climate phenomena such as El Niño and La Niña. These capabilities are supported by clear visualization and statistical test functions. Applying the refined parameters of CDM through PhenoFECT improved predictive performance by 1–8 days in RMSE for three representative model plants. In addition, the embedded dataset provides comprehensive phenological and meteorological records from South Korea and Japan, making the tool immediately applicable. PhenoFECT is also applicable to diverse user‐defined species of interest, including herbaceous plants such as dandelion (Appendix S4).

**Table 4:** Comparison of PhenoFECT's capabilities, model types, optimization strategy, scope, and embedded dataset against well‐established phenology modeling tools.

Tools	Capabilities	Model types	Optimization strategy	Scope	Embedded dataset	Supporting reference
PhenoFECT	Site‐ and species‐specific CDM refinement and integrated exploration workflow	CDM	Grid search	Orchard crops and deciduous forest trees	>50 years of phenological data for 9 species and daily meteorological recordings in East Asia	Current study
pyPhenology	Common Python framework for diverse phenology models	13 types (10 primary and 3 ensemble)	Differential evolution, basin hopping, brute force	Dependent on model types	~10 years of phenological data for 2 species and daily temperature recordings in the United States	Taylor (2018)
PhenoForecaster	MFD prediction for angiosperms without observation	Elastic net regression	ElasticNetCV	2320 angiosperm species	None	Park et al. (2019)
ChillR	Chilling and heat unit calculation and PLS analysis for bloom dates	Chilling hours, Utah chill units, growing degree hours	Simulated annealing algorithm	Temperate fruit trees	25 years of cherry flowering data and daily temperature recordings in Germany	Luedeling et al. (2025)

*Note*: CDM, chill‐day model; MFD, mean flowering date; PLS, partial least squares.

Beyond the convenience of analyzing climate change indicators by predicting the phenological events of plants, PhenoFECT is widely adaptable; for example, the program offers the ability to expand temperature‐based models by introducing additional meteorological parameters such as daylength, suggesting photoperiod (expressed as *L*_*c*_) as a potential parameter. Importantly, combining photoperiods improved predictions under highly variable weather conditions such as mild winters, which present challenges for temperature‐based models. Although recent AI‐based models achieve comparable or better predictive accuracy (Garnot et al., 2025), the extension of the CDM with a photoperiod‐related parameter demonstrates the possibility of refining mechanistic models while preserving biological interpretability.

Unique regional characteristics or nationwide climate patterns where the prediction of phenological event dates diverge from actual observations can be classified using hierarchical clustering to refined model outputs. From this perspective, the generalizability of PhenoFECT could be further evaluated by applying it to the phenology of Arctic plants, where climate change is progressing most rapidly, but its effects remain poorly studied (Panchen et al., 2025).

## AUTHOR CONTRIBUTIONS

I.‐G.C. conceived of the study and supervised it. S.W.K. collected open meteorological and phenology data. S.W.K. developed the Python package PhenoFECT and analyzed the Korean open datasets. S.W.K. drafted the manuscript, which was critically revised by I.‐G.C. Both authors read and approved the final manuscript.

## Supporting information


**Appendix S1:** Parameter estimation procedure for three spring‐flowering angiosperm species.

**Appendix S2:** Visualization of predictive accuracy of the refined chill‐day model on a regional scale using line graphs.

**Appendix S3:** Sensitivity of t‐distributed stochastic neighbor embedding (t‐SNE) visualization to random state and perplexity hyperparameters.

**Appendix S4:** Sample parameter estimation procedure for a herbaceous plant.

## Data Availability

The PhenoFECT source code, detailed user guide, and the embedded dataset used to demonstrate its utility are available at https://github.com/SongWon03/PhenoFECT.

## References

[aps370088-bib-0001] Cesaraccio, C. , D. Spano , R. L. Snyder , and P. Duce . 2004. Chilling and forcing model to predict bud‐burst of crop and forest species. Agricultural and Forest Meteorology 126: 1–13.

[aps370088-bib-0002] Cha, E. J. , J. G. Jhun , and H. S. Chung . 1999. A study on characteristics of climate in South Korea for El Niño/La Niña years. Journal of the Korean Meteorological Society 35: 98–117.

[aps370088-bib-0003] Doi, H. , and I. Katano . 2008. Phenological timings of leaf budburst with climate change in Japan. Agricultural and Forest Meteorology 148: 512–516.

[aps370088-bib-0004] Everitt, B. S. , and G. Dunn . 2001. Cluster analysis. In B. S. Everitt and G. Dunn [eds.], Applied multivariate data analysis, 125–160. Wiley, Chichester, United Kingdom.

[aps370088-bib-0005] Garnot, V. S. F. , L. Spafford , J. Lever , C. Sigg , B. Pietragalla , Y. Vitasse , A. Gessler , and J. D. Wegner . 2025. Deep learning meets tree phenology modelling: PhenoFormer versus process‐based models. Methods in Ecology and Evolution 16: 1489–1506.

[aps370088-bib-0006] Gilreath, P. R. , and D. W. Buchanan . 1981. Rest prediction model for low‐chilling ‘Sungold’ nectarine. Journal of the American Society for Horticultural Science 106: 426–429.

[aps370088-bib-0007] Ho, C.‐H. , E.‐J. Lee , I. Lee , and S.‐J. Jeong . 2006. Earlier spring in Seoul, Korea. International Journal of Climatology 26: 2117–2127.

[aps370088-bib-0008] Jung, J. E. , E. Y. Kwon , U. Chung , and J. I. Yun . 2005. Predicting cherry flowering date using a plant phonology model. Korean Journal of Agricultural and Forest Meteorology 7: 148–155.

[aps370088-bib-0009] Kim, J. H. , E.‐J. Lee , and J. I. Yun . 2013. Prediction of blooming dates of spring flowers by using digital temperature forecasts and phenology models. Korean Journal of Agricultural and Forest Meteorology 15: 40–49.

[aps370088-bib-0010] Korea Meteorological Administration (KMA) . 2016. Seasonal observation guidelines. Website https://data.kma.go.kr/data/publication/publicationGlList.do?ppgmNo=681 [accessed 19 March 2025].

[aps370088-bib-0011] Korea Meteorological Administration (KMA) . 2025a. ASOS. Website https://data.kma.go.kr/data/grnd/selectAsosRltmList.do?pgmNo=36 [accessed 1 May 2025].

[aps370088-bib-0012] Korea Meteorological Administration (KMA) . 2025b. ENSO occurrence status. Website http://www.climate.go.kr/home/05_prediction_new/predict02_02.html [accessed 29 August 2025].

[aps370088-bib-0013] Korea Meteorological Administration (KMA) . 2025c. Seasonal observation data. Website https://data.kma.go.kr/data/seasonObs/seasonObsDataList.do?pgmNo=648 [accessed 11 April 2025].

[aps370088-bib-0014] Körner, C. , and D. Basler . 2010. Phenology under global warming. Science 327: 1461–1462. 10.1126/science.1186473 20299580

[aps370088-bib-0015] Lee, S. H. , and K. M. Lee . 2003. The trend on the change of the cherry blossom flowering time due to the temperature change. Journal of Environmental Impact Assessment 12: 45–54.

[aps370088-bib-0016] Lee, H.‐S. , J.‐H. Kim , and J. I. Yun . 2014. Recent trends in blooming dates of spring flowers and the observed disturbance in 2014. Korean Journal of Agricultural and Forest Meteorology 16: 396–402.

[aps370088-bib-0017] Luedeling, E. , L. Caspersen , and E. Fernandez . 2025. chillR: Statistical methods for phenology analysis in temperate fruit trees. R package version 0.77. Website https://CRAN.R-project.org/package=chillR [accessed 6 May 2026].

[aps370088-bib-0018] Masson‐Delmotte, V. , P. Zhai , H.‐O. Pörtner , D. Roberts , J. Skea , P. R. Shukla , A. Pirani , et al. 2018. Global warming of 1.5°C. An IPCC Special Report on the impacts of global warming of 1.5°C above pre‐industrial levels and related global greenhouse gas emission pathways, in the context of strengthening the global response to the threat of climate change, sustainable development, and efforts to eradicate poverty. Intergovernmental Panel on Climate Change (IPCC). Cambridge University Press, Cambridge, United Kingdom.

[aps370088-bib-0019] Oh, S. D. , S. M. Kang , D. I. Kim , M. S. Kim , and W. S. Kim . 2004. Fruit tree physiology in relation to temperature. Gilmogeum, Seoul, Korea.

[aps370088-bib-0020] Panchen, Z. A. , J. Doubt , J. M. Saarela , and H. M. Kharouba . 2025. Digitised herbarium specimen data reveal a climate change‐related trend to an earlier, shorter Canadian Arctic flowering season, and phylogenetic signal in Arctic flowering times. New Phytologist 251: 696–706. 10.1111/nph.70386 PMC13278663 40654150

[aps370088-bib-0021] Park, I. , A. Jones , and S. J. Mazer . 2019. PhenoForecaster: A software package for the prediction of flowering phenology. Applications in Plant Sciences 7: e1230. 10.1002/aps3.1230 PMC6426163 30937222

[aps370088-bib-0022] Parolini, G. 2022. Weather, climate, and agriculture: Historical contributions and perspectives from agricultural meteorology. WIREs Climate Change 13: e766.

[aps370088-bib-0023] Richardson, E. A. , S. D. Seeley , and D. R. Walker . 1974. A model for estimating the completion of rest for Redhaven and Elberta peach trees. HortScience 9: 331–332.

[aps370088-bib-0024] Taylor, S. D. 2018. pyPhenology: A python framework for plant phenology modeling. Journal of Open Source Software 3: e827.

[aps370088-bib-0025] Uryu, S. 2024. jmastats: Download weather data from Japan Meteorological Agency. R package version 0.2.1. Website https://CRAN.R-project.org/package=jmastats [accessed 12 January 2026].

[aps370088-bib-0026] Ward, J. H. 1963. Hierarchical grouping to optimize an objective function. Journal of the American Statistical Association 58: 236–244.

