# Supplementary material for: PhenoFECT: A Python package for forecasting and exploring plant phenology under climate change
Source: Appl Plant Sci. 2026 Oct 30:e70088. Online ahead of print. doi: 10.1002/aps3.70088 (PMC13722615; doi:10.1002/aps3.70088)
Supplement: Supplementary file 1 [file APS3-9999-e70088-s001.docx]

**Appendix S2.** Visualization of predictive accuracy of the refined chill-day model on a regional scale using line graphs.

**Visualization of predictive accuracy of the refined model for cherry (*Prunus yedoensis*)**

Users can plot a line graph for user-specified locations. The *comparing_linegraph* function requires a list of observatory stations and phenological observatory data. Here, we show the line graphs for the two regions with the lowest RMSE.

\# For visualization of the best fit parameters

target_location = ['Gumi', 'Cheongju']

best_fit = "ChillDayModel_bestfit, Tc_3.9, Cr_-108, Hr_164.csv”

phenology_visualization.comparing_linegraph(best_fit_df, best_fit, phenological_data, target_location)

**Visualization of predictive accuracy of the refined model for** **golden-bell (*Forsythia koreana*)**

We changed only the list of observatory stations as ‘target_location = ['Gwangju', 'Chuncheon']’.

\# For visualization of the best fit parameters

target_location = ['Gwangju', 'Chuncheon']

best_fit = “ChillDayModel_bestfit, Tc_3.2, Cr_-81, Hr_132.csv”

phenology_visualization.comparing_linegraph(best_fit_df, best_fit, phenological_data, target_location)

**Visualization of predictive accuracy of the refined model for azalea (*Rhododendron mucronulatum*)**

We changed only the list of observatory stations as ‘target_location = ['Chuncheon', 'Cheongju']’.

\# For visualization of the best fit parameters

target_location = ['Chuncheon', ‘Cheongju’]

best_fit = “ChillDayModel_bestfit, Tc_2.8, Cr_-68, Hr_158.csv”

phenology_visualization.comparing_linegraph(best_fit_df, best_fit, phenological_data, target_location)
