# Supplementary material for: PhenoFECT: A Python package for forecasting and exploring plant phenology under climate change
Source: Appl Plant Sci. 2026 Oct 30:e70088. Online ahead of print. doi: 10.1002/aps3.70088 (PMC13722615; doi:10.1002/aps3.70088)
Supplement: Supplementary file 2 [file APS3-9999-e70088-s002.docx]

**Appendix S3.** Sensitivity of t-distributed stochastic neighbor embedding (t-SNE) visualization to random state and perplexity hyperparameters.

The values of ‘random_states = 150’ and ‘perplexity = 50’ were used as a default in the *tsne_visualization* function argument. Here, we compare the four plots generated by four different hyperparameter combinations.

With the values ‘random_states = 50’ and ‘perplexity = 10’, the t-SNE plot was visualized as shown in the left panel of the figure below. With the values ‘random_states = 100’ and ‘perplexity = 10’, the t-SNE plot was visualized as shown in the right panel of the figure below. The global pattern of visualization was almost reproduced when different ‘random_states’ values were tested.

With the values ‘random_states = 50’ and ‘perplexity = 30’, the t-SNE plot was visualized as shown in the left panel of the figure below. The global pattern of visualization was quite changed when different ‘perplexity’ values were tested because the higher value considers a relatively large number of nearest neighbors in manifold learning. With the values ‘random_states = 100’ and ‘perplexity = 30’, the t-SNE plot was visualized as shown in the right panel of the figure below. The global pattern of visualization was almost reproduced when different ‘random_states’ values for the same ‘perplexity’ were tested except for the cluster 9 (gold).
