# Supplementary material for: PhenoFECT: A Python package for forecasting and exploring plant phenology under climate change
Source: Appl Plant Sci. 2026 Oct 30:e70088. Online ahead of print. doi: 10.1002/aps3.70088 (PMC13722615; doi:10.1002/aps3.70088)
Supplement: Supplementary file 3 [file APS3-9999-e70088-s003.docx]

**Appendix S4.** Sample parameter estimation procedure for a herbaceous plant.

**Estimation of parameter set for dandelion (*Taraxacum*)**

The phenology dataset for dandelion was split in three training and test datasets by random sampling per each decade starting from 1952. Because there were only records of flowering events for dandelions, we searched the parameter space without constraining the range in step 1. Phenological events were observed from 1952 to 2020, and we used the 53 locations where more than 80% of observations exist.

Step 1: The *predict_flowering* function was applied to the diverse parameter combinations. At the first round of grid search, the 6798 combinations of temperature threshold (*T_c_*), chilling requirement (*C_r_*), and heat requirement (*H_r_*) within the ranges of 7–12°C (in 1°C increments), –100 to –199 (in 3-unit increments), and 110 to 248 (in 3-unit increments), respectively, were examined for each three training datasets.

[Codes for flowering prediction and heatmap visualization with simplified argument]

\# For flowering prediction

temperature_threshold_list = [tc for tc in range(7, 13, 1)]

chill_requirement_list = [cr for cr in range(–100, –200, –3)]

heat_requirement_list = [hr for hr in range(110, 250, 3)]

error_df = phenology_prediction.predict_flowering(meteorological_data, phenological_data, location_list, temperature_threshold_list, chill_requirement_list, heat_requirement_list, 1952, 2020)

\# For heatmap

phenology_visualization.flowering_error_heatmap(error_df, errortype = ‘RMSE’, annot =False, para1 = ‘temperature_threshold’, para2 = ‘chill_requirement’, figure_title = "Heatmap of RMSE Trend about Tc, Cr in Dandelion Flowering Prediction")

phenology_visualization.flowering_error_heatmap(error_df, errortype = ‘RMSE’, annot =False, para1 = ‘chill_requirement’, para2 = ‘heat_requirement’, figure_title = "Heatmap of RMSE Trend about Cr, Hr in Dandelion Flowering Prediction")

Figure A1. Output heatmaps of RMSE trends for *T_c_*, *C_r_*, and *H_r_* in dandelion flowering prediction.

The *flowering_error_heatmap* function visualizes the average RMSE value calculated from all 53 locations in the dataset where observations exist for more than 80% of recorded years. In the upper figure, the heatmap shows some empty grids due to the definition of *H_r_* that the heat requirement for flowering must be greater than the absolute *C_r_* value. The output heatmaps for the three training datasets were omitted in order to reduce redundancy. The best parameter sets for step 2 for each split dataset were *T_c_* = 7.0°C, *C_r_* = –100, and *H_r_* = 110 for all three training sets.

Step 2: Using the best-fit parameters in step 2 as the starting point, we applied a finer grid for each training dataset. We searched the 8379 combinations of *T_c_*, *C_r_*, and *H_r_* with an interval of 0.1°C for *T_c_* and 1 unit for *C_r_* and *H_r_* for each training set.

[Codes for flowering prediction and contour map with simplified argument]

\# For flowering prediction

temperature_threshold_list = [6.1, …, 7.0, …, 7.9]

chill_requirement_list = [cr for cr in range(–90, –111, –1)]

heat_requirement_list = [hr for hr in range(100, 121, 1)]

error_df = phenology_prediction.predict_flowering(meteorological_data, phenological_data, location_list, temperature_threshold_list, chill_requirement_list, heat_requirement_list, 1952, 2020)

\# For contourmap

phenology_visualization.flowering_error_contourmap(error_df, errortype = ‘RMSE’, para1 = ‘temperature_threshold’, para2 = ‘chill_requirement’, label1 = ‘Temperature threshold (Tc)’, label2 = ‘Chill requirement (Cr)’, label3 = “Contour Map of Minimum Root Mean Square Error (RMSE) for Tc & Cr combinations”)

phenology_visualization.flowering_error_contourmap(error_df, errortype = ‘RMSE’, para1 = ‘chill_requirement’, para2 = ‘heat_requirement’, label1 = ‘Chill requirement (Cr)’, label2 = ‘Heat requirement (Hr)’, label3 = “Contour Map of Minimum Root Mean Square Error (RMSE) for Cr & Hr combinations”)

Figure A2. Output contour maps of RMSE trends for *T_c_*, *C_r_*, and *H_r_* in dandelion flowering prediction.

The *flowering_error_contourmap* function similarly visualizes the average RMSE value calculated from all 53 locations. However, the visualized contour map demonstrates that the tested range of chill-day model (CDM) parameters was still not sufficient. We therefore went back to step 1 and searched another range of parameter space.

Step 3: In the third round of grid search, the 2436 combinations of *T_c_*, *C_r_*, and *H_r_* within the ranges of 2–5°C (in 1°C increments), –50 to –150 (in 5-unit increments), and 60 to 200 (in 5-unit increments), respectively, were examined for each of the three training datasets.

[Codes for flowering prediction and heatmap visualization with simplified argument]

\# For flowering prediction

temperature_threshold_list = [tc for tc in range(2, 6, 1)]

chill_requirement_list = [cr for cr in range(–50, –151, –5)]

heat_requirement_list = [hr for hr in range(60, 201, 5)]

error_df = phenology_prediction.predict_flowering(meteorological_data, phenological_data, location_list, temperature_threshold_list, chill_requirement_list, heat_requirement_list, 1952, 2020)

\# For heatmap

phenology_visualization.flowering_error_heatmap(error_df, errortype = ‘RMSE’, annot =False, para1 = ‘temperature_threshold’, para2 = ‘chill_requirement’, figure_title = "Heatmap of RMSE Trend about Tc, Cr in Dandelion Flowering Prediction")

phenology_visualization.flowering_error_heatmap(error_df, errortype = ‘RMSE’, annot =False, para1 = ‘chill_requirement’, para2 = ‘heat_requirement’, figure_title = "Heatmap of RMSE Trend about Cr, Hr in Dandelion Flowering Prediction")

Figure A3. Output heatmaps of RMSE trends for *T_c_*, *C_r_*, and *H_r_* in dandelion flowering prediction. The output heatmaps for the three training datasets were omitted in order to reduce redundancy.

The best parameter sets for step 3 for each split dataset were *T_c_* = 2.0°C, *C_r_* = –75, *H_r_* = 125 for training set 1, *T_c_* = 2.0°C, *C_r_* = –80, *H_r_* = 110 for training set 2, and *T_c_* = 2.0°C, *C_r_* = –50, *H_r_* = 165 for training set 3.

Step 4: In the fourth round of grid search, the 8379 combinations of *T_c_*, *C_r_*, and *H_r_* with the resolution of 0.1°C increments, 1-unit increments, and 1-unit increments, respectively, for each three best parameters in step 3. Sample codes for training set 2 and 3 were omitted to reduce redundancy.

[Codes for flowering prediction and contour map with simplified argument]

\# For flowering prediction (only training set 1 was explored with two cases of parameter combinations)

\# Case 1

temperature_threshold_list = [1.1, …, 2.0, …, 2.9]

chill_requirement_list = [cr for cr in range(–65, –86, –1)]

heat_requirement_list = [hr for hr in range(115, 136, 1)]

\# Case 2

temperature_threshold_list = [1.1, …, 2.0, …, 2.9]

chill_requirement_list = [cr for cr in range(–40, –61, –1)]

heat_requirement_list = [hr for hr in range(155, 176, 1)]

error_df = phenology_prediction.predict_flowering(meteorological_data, phenological_data, location_list, temperature_threshold_list, chill_requirement_list, heat_requirement_list, 1952, 2020)

\# For contourmap

phenology_visualization.flowering_error_contourmap(error_df, errortype = ‘RMSE’, para1 = ‘temperature_threshold’, para2 = ‘chill_requirement’, label1 = ‘Temperature threshold (Tc)’, label2 = ‘Chill requirement (Cr)’, label3 = “Contour Map of Minimum Root Mean Square Error (RMSE) for Tc & Cr combinations”)

phenology_visualization.flowering_error_contourmap(error_df, errortype = ‘RMSE’, para1 = ‘chill_requirement’, para2 = ‘heat_requirement’, label1 = ‘Chill requirement (Cr)’, label2 = ‘Heat requirement (Hr)’, label3 = “Contour Map of Minimum Root Mean Square Error (RMSE) for Cr & Hr combinations”)

Figure A4. Output contour maps of RMSE trends for *T_c_*, *C_r_*, and *H_r_* in dandelion flowering prediction for training set 1.

The best-fit parameter set that best explains phenological events of dandelion in step 4 was *T_c_* = 1.3°C, *C_r_* = –44, and *H_r_* = 155 for training set 1, *T_c_* = 2.0°C, *C_r_* = –80, and *H_r_* = 111 for training set 2, and *T_c_* = 1.4°C, *C_r_* = –40, and *H_r_* = 171 for training set 3.

The optimal parameter set was selected by cross-validation of each best-fit parameter set to all the three test datasets. The optimal parameter set was defined as the parameter set with the minimum average of the three RMSE values. The optimal parameter set that best explains phenological events of dandelion (*Taraxacum*) was *T_c_* = 1.4°C, *C_r_* = –40, and *H_r_* = 171, with an average RMSE of 14.71 for the three test datasets. Although there were 16 locations with an RMSE of less than 10 days, the flowering dates for 18 locations were not predicted.
