# Supplementary material for: PhenoFECT: A Python package for forecasting and exploring plant phenology under climate change
Source: Appl Plant Sci. 2026 Oct 30:e70088. Online ahead of print. doi: 10.1002/aps3.70088 (PMC13722615; doi:10.1002/aps3.70088)
Supplement: Supplementary file 4 [file APS3-9999-e70088-s004.docx]

**Appendix S1.** Parameter estimation procedure for three spring-flowering angiosperm species.

**Estimation of parameter set for cherry (*Prunus yedoensis*)**

The phenology dataset for each angiosperm species was split into three training and test datasets by random sampling per each decade starting from 1973. In step 1, we utilized only the first dataset.

Step 1: To constrain the range of parameter space for flowering date, 1200 combinations of temperature threshold (*T_c_*) and chilling requirement (*C_r_*) within the ranges of 2–8°C (in 1°C increments) and –50 to -200 (in 1-unit increments), respectively, were examined.

[Codes for budburst prediction and heatmap visualization with simplified argument]

\# For bud-burst prediction

temperature_threshold_list = [tc for tc in range(2, 9, 1)]

chill_requirement_list = [cr for cr in range(–50, –201, –1)]

rmse_df = phenology_prediction.predict_budburst(meteorological_data, phenological_data, location_list, temperature_threshold_list, chill_requirement_list, 1973, 2025)

\# For visualization

phenology_visualization.budburst_error_heatmap(rmse_df, arrange = 1, annot=False, figure_title = “Heatmap of RMSE Trend about Tc, Cr in Cherry Bud-burst Prediction")

\# For selecting parameter range belonged to first quartile (Q1) of RMSE

phenology_prediction.search_space_extractor(rmse_df, rmse_column = “RMSE”, tc_column = “temperature_threshold”, cr_column = “chill_requirement”, q1_quantile = 0.25, verbose = True)

The annotations for root mean square error (RMSE) value were not represented in the output heatmap to maintain visual simplicity and clarity. Users can select whether or not to visualize the annotation of RMSE value with the “annot = True” argument in the *budburst_error_heatmap* function. The *search_space_extractor* function was applied to compute the parameter range belonging to the first quartile (Q1) of RMSE.

Figure A1. Output heatmap of RMSE trends for *T_c_* and *C_r_* in cherry budburst prediction.

Step 2: The *predict_flowering* function was applied to the range of parameter combinations that belonged to the first quartile (Q1) of RMSE in the budburst prediction. At the first round of grid search, the 4968 combinations of *T_c_*, *C_r_*, and heat requirement (*H_r_*) within the ranges of 2–7°C (in 1°C increments), –67 to –133 (in 3-unit increments), and 77 to 182 (in 3-unit increments), respectively, were examined for each of the three training datasets.

[Codes for flowering prediction and heatmap visualization with simplified argument]

\# For flowering prediction

temperature_threshold_list = [tc for tc in range(2, 8, 1)]

chill_requirement_list = [cr for cr in range(–67, –134, –3)]

heat_requirement_list = [hr for hr in range(77, 184, 3)]

error_df = phenology_prediction.predict_flowering(meteorological_data, phenological_data, location_list, temperature_threshold_list, chill_requirement_list, heat_requirement_list, 1973, 2025)

\# For heatmap

phenology_visualization.flowering_error_heatmap(error_df, errortype = ‘RMSE’, annot =True, para1 = ‘temperature_threshold’, para2 = ‘chill_requirement’, figure_title = "Heatmap of RMSE Trend about Tc, Cr in Cherry Flowering Prediction")

phenology_visualization.flowering_error_heatmap(error_df, errortype = ‘RMSE’, annot =False, para1 = ‘chill_requirement’, para2 = ‘heat_requirement’, figure_title = "Heatmap of RMSE Trend about Cr, Hr in Cherry Flowering Prediction")

Figure A2. Output heatmaps of RMSE trends for *T_c_*, *C_r_*, and *H_r_* in cherry flowering prediction.

The *flowering_error_heatmap* function visualizes the average RMSE value calculated from all 32 locations in the dataset where observations exist for more than 80% of recorded years. In the upper figure, the heatmap shows some empty grids due to the definition of *H_r_* that the heat requirement for flowering must be greater than the absolute value of *C_r_*. The output heatmaps for the three training datasets were omitted to reduce redundancy. The best parameter sets for step 2 for each split dataset were *T_c_* = 4.0°C, *C_r_* = –109, *H_r_* = 161 for set 1, *T_c_* = 5.0°C, *C_r_* = –127, *H_r_* = 134 for set 2, and *T_c_* = 4.0°C, *C_r_* = –112, *H_r_* = 161 for set 3.

Step 3: Using the previously best fit parameters in step 2 as the starting point, we applied a finer grid for each training dataset. We searched the 8379 combinations *T_c_*, *C_r_*, and *H_r_* with an interval of 0.1°C for *T_c_* and 1 unit for *C_r_* and *H_r_* for each training set.

[Codes for flowering prediction and contour map with simplified argument]

\# For flowering prediction

temperature_threshold_list = [3.1, …, 4.0, …, 4.9]

chill_requirement_list = [cr for cr in range(–99, –120, –1)]

heat_requirement_list = [hr for hr in range(151, 172, 1)]

error_df = phenology_prediction.predict_flowering(meteorological_data, phenological_data, location_list, temperature_threshold_list, chill_requirement_list, heat_requirement_list, 1973, 2025)

\# For contourmap

phenology_visualization.flowering_error_contourmap(error_df, errortype = ‘RMSE’, para1 = ‘temperature_threshold’, para2 = ‘chill_requirement’, label1 = ‘Temperature threshold (Tc)’, label2 = ‘Chill requirement (Cr)’, label3 = “Contour Map of Minimum Root Mean Square Error (RMSE) for Tc & Cr combinations”)

phenology_visualization.flowering_error_contourmap(error_df, errortype = ‘RMSE’, para1 = ‘chill_requirement’, para2 = ‘heat_requirement’, label1 = ‘Chill requirement (Cr)’, label2 = ‘Heat requirement (Hr)’, label3 = “Contour Map of Minimum Root Mean Square Error (RMSE) for Cr & Hr combinations”)

Figure A3. Output contour maps of RMSE for *T_c_*, *C_r_*, and *H_r_* in cherry flowering prediction.

The *flowering_error_contourmap* function similarly visualizes the average RMSE value calculated from all 32 locations. The best-fit parameter set that best explains phenological events of cherry in step 3 was *T_c_* = 4.2°C, *C_r_* = –114, and *H_r_* = 158 for training set 1, *T_c_* = 4.8°C, *C_r_* = –121, and *H_r_* = 140 for training set 2, and *T_c_* = 3.9°C, *C_r_* = –108, and *H_r_* = 164 for training set 3.

The optimal parameter set was selected by cross-validation of each best-fit parameter set to all the three test datasets. The optimal parameter set was defined as the parameter set with the minimum average of the three RMSE values. The optimal parameter set that best explains phenological events of cherry (*Prunus yedoensis*) was *T_c_* = 3.9°C, *C_r_* = –108, and *H_r_* = 164 with 4.61 of average RMSE for the three test datasets. Note that the minimum RMSE difference calculated by changing one unit range of the optimal parameter set was less than 0.1. This means that users can ignore an RMSE difference of less than 0.1 when estimating chill-day model (CDM) parameters using the same resolution used in step 3 for cherry phenology prediction.

**Estimation of parameter set for golden-bell (*Forsythia koreana*)**

Step 1: To constrain the range of parameter space for flowering date, 1200 combinations of *T_c_* and *C_r_* within the ranges of 2–8°C (in 1°C increments) and –50 to -200 (in 1-unit increments), respectively, were examined. The same codes are used to refine the CDM for golden-bell, except for the kind of phenological data and labeling in the function arguments.

[Codes for heatmap of budburst prediction result with simplified argument]

\# For visualization

phenology_visualization.budburst_error_heatmap(rmse_df, arrange = 2, figure_title = “Heatmap of RMSE Trend about Tc, Cr in Golden-bell Bud-burst Prediction")

Figure A4. Heatmap of RMSE trends for *T_c_* and *C_r_* in *Forsythia koreana* budburst prediction.

Users can change the axis of ‘temperature_threshold’ and ‘chill_requirement’ using the *budburst_error_heatmap* function argument ‘arrange’ as 2.

Step 2: The *predict_flowering* function was applied to the range of parameter combinations that belonged to the first quartile (Q1) of RMSE in the budburst prediction. At the first round of grid search, the 5328 combinations of *T_c_*, *C_r_*, and *H_r_* within the ranges of 2–7°C (in 1°C increments), –53 to –122 (in 3-unit increments), and 63 to 171 (in 3-unit increments), respectively, were examined for each of the three training datasets.

[Codes for heatmap of flowering prediction result with simplified argument]

\# For heatmap

phenology_visualization.flowering_error_heatmap(error_df, errortype = ‘MAE’, annot =True, para1 = ‘temperature_threshold’, para2 = ‘chill_requirement’, figure_title = "Heatmap of MAE Trend about Tc, Cr in Forsythia Flowering Prediction")

phenology_visualization.flowering_error_heatmap(error_df, errortype = ‘MAE’, annot =False, para1 = ‘chill_requirement’, para2 = ‘heat_requirement’, figure_title = "Heatmap of MAE Trend about Cr, Hr in Forsythia Flowering Prediction")

Figure A5. Output heatmaps of RMSE trends for *T_c_*, *C_r_*, and *H_r_* in *Forsythia koreana* flowering prediction.

Users can select the error type of the prediction with the examined parameter combinations using both RMSE and mean absolute error (MAE) by setting the *flowering_error_heatmap* function argument ‘error_type’ to either ‘RMSE’ or ‘MAE’.

The output heatmaps for the three training datasets were omitted in order to reduce redundancy. The best parameter sets for step 2 for each split dataset were *T_c_* = 3.0°C, *C_r_* = –74, *H_r_* = 138 for set 1, *T_c_* = 3.0°C, *C_r_* = –77, *H_r_* = 138 for set 2, and *T_c_* = 3.0°C, *C_r_* = –74, *H_r_* = 141 for set 3.

Step 3: Using the best-fit parameters in step 2 as the starting point, we applied a finer grid for each training dataset. We searched the 8379 combinations *T_c_*, *C_r_*, and *H_r_* with an interval of 0.1°C for *T_c_* and 1 unit for *C_r_* and *H_r_* for each training set.

Figure A6. Output contour maps of RMSE for *T_c_*, *C_r_*, and *H_r_* in *Forsythia koreana* flowering prediction.

The *flowering_error_contourmap* function similarly visualizes the average RMSE value calculated from all 31 locations. The best-fit parameter set that best explains phenological events of *Forsythia* *koreana* in step 3 was *T_c_* = 3.2°C, *C_r_* = –81, and *H_r_* = 132 for training set 1, *T_c_* = 2.9°C, *C_r_* = –71, and *H_r_* = 148 for training set 2, and *T_c_* = 2.9°C, *C_r_* = –71, and *H_r_* = 144 for training set 3.

The optimal parameter set was selected by cross-validation of each best-fit parameter set to the three test datasets. The optimal parameter set was defined as the parameter set with the minimum average RMSE values. The optimal parameter set that best explains the phenological events of golden-bell (*Forsythia koreana*) was *T_c_* = 3.2°C, *C_r_* = –81, and *H_r_* = 132, with an average RMSE of 5.96 for the three test datasets. Note that the minimum RMSE difference calculated by changing one unit range of the optimal parameter set was less than 0.1. This means that users can ignore an RMSE difference of less than 0.1 when estimating CDM parameters using the same resolution as in step 3 for *Forsythia* *koreana* phenology prediction.

**Estimation of parameter set for azalea (*Rhododendron mucronulatum*)**

Step 1: To constrain the range of parameter space for flowering date, 1200 combinations of *T_c_* and *C_r_* within the ranges of 2–8°C (in 1°C increments) and –50 to -200 (in 1-unit increments), respectively, were examined. The same codes are used to refine the CDM for azalea, except for the kind of phenological data and labeling in the function arguments. A total of 26 locations were used due to a significant amount of missing data at some sites.

[Codes for heatmap of budburst prediction result with simplified argument]

\# For visualization

phenology_visualization.budburst_error_heatmap(rmse_df, arrange = 1, figure_title = “Heatmap of RMSE Trend about Tc, Cr in Azalea Bud-burst Prediction")

Figure A7. Heatmap of RMSE trends for *T_c_* and *C_r_* in azalea budburst prediction.

Step 2: The *predict_flowering* function was applied to the range of parameter combinations that belonged to the first quartile (Q1) of RMSE in the budburst prediction. In the first round of grid search, the 4968 combinations of *T_c_*, *C_r_*, and *H_r_* within the ranges of 2–7°C (in 1°C increments), –55 to –121 (in 3-unit increments), and 65 to 170 (in 3-unit increments), respectively, were examined for each of the three training datasets.

The same codes are used to refine the CDM for azalea, and we used RMSE heatmap to visualize the error trend of the tested CDM parameters.

Figure A8. Output heatmaps of RMSE trends for *T_c_*, *C_r_*, and *H_r_* in azalea flowering prediction.

Step 3: Using the best-fit parameters in step 2 as the starting point, we applied a finer grid for each training dataset. We searched the 8379 combinations of *T_c_*, *C_r_*, and *H_r_* with an interval of 0.1°C for *T_c_* and 1 unit for *C_r_* and *H_r_* for each training set.

Figure A9. Output contour maps of RMSE trends for *T_c_*, *C_r_*, and *H_r_* in azalea flowering prediction.

The *flowering_error_contourmap* function similarly visualizes the average RMSE value calculated from all 31 locations. The best-fit parameter set that best explains phenological events of azalea in step 3 was *T_c_* = 2.8°C, *C_r_* = –68, and *H_r_* = 158 for all three training sets.

The optimal parameter set that best explains phenological events of azalea (*Rhododendron mucronulatum*) was *T_c_* = 2.8°C, *C_r_* = –68, and *H_r_* = 158 with an average RMSE of 6.66 for the three test datasets. Note that the minimum RMSE difference calculated by changing one unit range of the optimal parameter set was less than 0.1. This means that users can ignore an RMSE difference of less than 0.1 when estimating CDM parameters using the same resolution as in step 3 for azalea phenology prediction.
